# Xyloglucan Is Not Essential for the Formation and Integrity of the Cellulose Network in the Primary Cell Wall Regenerated from *Arabidopsis* Protoplasts

**DOI:** 10.3390/plants9050629

**Published:** 2020-05-14

**Authors:** Hiroaki Kuki, Ryusuke Yokoyama, Takeshi Kuroha, Kazuhiko Nishitani

**Affiliations:** 1 Department of Developmental Biology and Neurosciences, Graduate School of Life Sciences, Tohoku University, Sendai 980-8578, Japan; kukihiro@mail.saitama-u.ac.jp (H.K.); ryusuke.yokoyama.d6@tohoku.ac.jp (R.Y.); kurohat236@affrc.go.jp (T.K.); 2 Division of Life Science, Graduate School of Science and Engineering, Saitama University, 255 Shimo-okubo, Sakura-ku, Saitama 338-8570, Japan; 3 Division of Applied Genetics, Institute of Agrobiological Sciences, National Agriculture and Food Research Organization (NARO), Tsukuba, Ibaraki 305-8604, Japan; 4 Department of Biological Science, Faculty of Science, Kanagawa University, 2946 Tsuchiya, Hiratsuka, Kanagawa 259-1293, Japan

**Keywords:** *Arabidopsis thaliana*, *xxt1 xxt2*, primary cell wall, cellulose microfibril, xyloglucan, protoplast, image analysis

## Abstract

The notion that xyloglucans (XG) play a pivotal role in tethering cellulose microfibrils in the primary cell wall of plants can be traced back to the first molecular model of the cell wall proposed in 1973, which was reinforced in the 1990s by the identification of Xyloglucan Endotransglucosylase/Hydrolase (XTH) enzymes that cleave and reconnect xyloglucan crosslinks in the cell wall. However, this tethered network model has been seriously challenged since 2008 by the identification of the *Arabidopsis thaliana* xyloglucan-deficient mutant (*xxt1 xxt2*), which exhibits functional cell walls. Thus, the molecular mechanism underlying the physical integration of cellulose microfibrils into the cell wall remains controversial. To resolve this dilemma, we investigated the cell wall regeneration process using mesophyll protoplasts derived from *xxt1 xxt2* mutant leaves. Imaging analysis revealed only a slight difference in the structure of cellulose microfibril network between *xxt1 xxt2* and wild-type (WT) protoplasts. Additionally, exogenous xyloglucan application did not alter the cellulose deposition patterns or mechanical stability of *xxt1 xxt2* mutant protoplasts. These results indicate that xyloglucan is not essential for the initial assembly of the cellulose network, and the cellulose network formed in the absence of xyloglucan provides sufficient tensile strength to the primary cell wall regenerated from protoplasts.

## 1. Introduction

Primary cell walls provide plant cells with the flexibility to expand and the mechanical strength to support the cell shape. The primary cell wall is mainly composed of cellulose microfibrils and matrix polysaccharides such as xyloglucan (XG) and pectin. While cellulose microfibrils are synthesized by the membrane-localized cellulose synthase complex [1,2], matrix polysaccharides are polymerized in the Golgi and secreted into the cell wall space, where an ordered molecular network structure is constructed using the cellulose microfibrils and the matrix polysaccharides [3,4].

The first molecular model for the network structure of the primary cell wall was proposed by Albersheim and colleagues [5]. In this model, xyloglucans were envisaged to tightly bind to cellulose microfibrils via hydrogen bonds, while other matrix polymers such as pectin were linked to xyloglucan via covalent bonds. This original model was supported by studies showing that xyloglucan dynamics is involved in cell wall construction and extension processes [6,7,8,9]. However, this model was later revised and replaced by the tethered network model, which suggests that a single xyloglucan molecule physically interacts with two or more cellulose microfibrils, thus functioning as a tether between microfibrils [4,10,11,12]. The tethered network model was reinforced by the identification of a xyloglucan endotransglucosylase/hydrolase, an enzyme that cleaves and reconnects xyloglucan crosslinks [13,14,15].

In 2008, the tethered network model was seriously challenged by the identification of *Arabidopsis thaliana* xyloglucan xylosyltransferase (XXT) double mutant, *xxt1 xxt2*, which lacks detectable xyloglucan [16]. Despite the absence of xyloglucan, the *xxt1 xxt2* double mutant shows normal cell wall architecture and exhibits only minor growth and developmental defects [16]. This finding questioned the role of xyloglucan in primary cell wall formation proposed in conventional models. Furthermore, solid-state nuclear magnetic resonance (NMR)-based studies showed that only a small proportion of the cellulose surface is in direct contact with xyloglucan in *Arabidopsis* cell walls [17,18,19,20,21], suggesting that xyloglucan is closely intertwined with cellulose at limited sites, referred to as biomechanical hot-spots, in the primary cell wall [22,23,24]. Although the *xxt1 xxt2* double mutant displayed relatively minor phenotypic defects, some studies showed that xyloglucan deficiency led to the disruption of the network mainly composed of cellulose microfibrils [25,26], resulting in the formation of a more fragile and extensible cell wall [16,27]. These results suggest that xyloglucan contributes to the physical properties of primary cell walls. However, whether and how the abnormality of the functional network structure is caused by xyloglucan deficiency has not yet been elucidated.

Cell wall regeneration from protoplasts is the only approach that could be used to directly observe the de novo construction of cell walls [28,29,30,31,32]. We recently developed a procedure to visualize cell wall formation from *Arabidopsis* mesophyll protoplasts using confocal laser scanning microscopy, and quantitatively analyzed several features such as the amount of the network and bundling of cellulose fibrils during cell wall formation using image analysis [33]. To determine the roles of xyloglucan in the cellulose network formation, we used this image analysis and analyzed the cell wall regeneration process in *xxt1 xxt2* double mutant protoplasts derived from mesophyll cells. We also investigated the effects of exogenously applied xyloglucan on the construction of cellulose network in the primary cell wall regenerated from *xxt1 xxt2* protoplasts.

## 2. Results

### 2.1. Comparative Analysis of Network Structure in Cell Walls Regenerated from xxt1 xxt2 and Wild-Type (WT) Protoplasts

**Figure 1:**
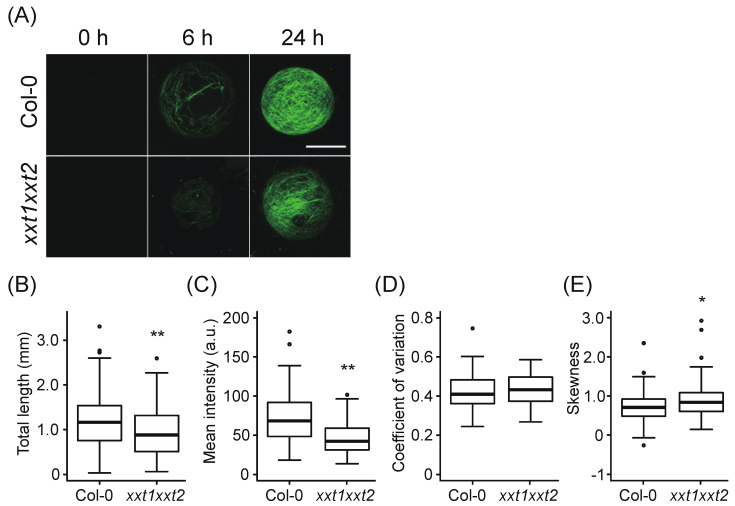
Comparison of cell wall regeneration between WT (Col-0) and *xxt1 xxt2* protoplasts. (**A**) Representative images of network structure in the cell wall of Col-0 and *xxt1 xxt2* protoplasts incubated for 0, 6, and 24 h and stained with Calcofluor. (**B**–**E**) Total length (**B**), mean intensity (**C**), coefficient of variation (CV) of fluorescence intensity distribution (**D**), and skewness of fluorescence intensity distribution (**E**) of the network measured at 24 h. Center lines of box-plot show the medians, boxes indicate interquartile range (IQR), whiskers indicate 1.5 IQR, and outliers are shown by dots. The experiment was repeated three times and summarized data was subjected to Mann–Whitney test (**, *p* < 0.01; *, 0.01 ≤ *p* < 0.05; *n* = 130 (Col-0) and 88 (*xxt1 xxt2*)). Scale bar = 20 μm.

We recently established an imaging technique to quantitatively evaluate the cell wall regeneration process from *Arabidopsis* mesophyll protoplasts [33]. Using this technique, we first compared cell wall regeneration processes between wild-type (WT) and *xxt1 xxt2* protoplasts. Mesophyll protoplasts isolated from WT and *xxt1 xxt2* leaves were incubated in cell wall regeneration medium and stained with Calcofluor, a β-glucan-specific dye. The results showed that fibrous structures were constructed on the surface of protoplasts within 6 h of incubation and developed further during 24 h of incubation (Figure 1A). To evaluate features of the regenerated cell wall network, we measured the total length, mean intensity, and bundling degree of the network in WT and *xxt1 xxt2* protoplasts incubated in the regeneration medium for 24 h. The total length of the fibrous structure was shorter in *xxt1 xxt2* protoplasts than in WT protoplasts (Figure 1B). In *xxt1 xxt2* protoplasts, the mean Calcofluor signal intensity decreased relative to WT protoplasts (Figure 1C). Since Calcofluor stains xyloglucan as well as cellulose [34], the decrease in the Calcofluor signal in *xxt1 xxt2* protoplasts probably reflects the deficiency of xyloglucan. It has been reported that the amount of cellulose deposition of *xxt1 xxt2* is 20% lower than that of the WT [26], and this is comparable to the difference in our measurement of total length (Figure 1B). Therefore, changes in the amount of cellulose could be measured by the total length rather than the mean fluorescence intensity.

We next measured the coefficient of variation (CV) and skewness of intensity distribution of Calcofluor signal in WT and *xxt1 xxt2* protoplasts. While there was no significant difference in the CV of Calcofluor signal between WT and *xxt1 xxt2* protoplasts (Figure 1D), skewness showed a slight but significant difference between protoplasts of the two genotypes (Figure 1E). To determine which of the two statistical measures (skewness or CV) was appropriate for determining the bundling of Calcofluor-stained fibers, we compared the skewness and CV of intensity distribution of cellulose fibrils in protoplasts treated with Taxol, an inhibitor of cortical microtubule depolymerization; we previously showed that Taxol causes bundling of cellulose fibrils in protoplasts [33]. The results showed that CV was a more sensitive metric for quantifying the bundling of the cellulose network than skewness, and was suitable for assessing the bundling of cellulose fibrils (Figure S1) (For the skewness metric, see [33]). Since the mean intensity and skewness showed a negative correlation, which was more prominent than the negative correlation between mean intensity and CV (Figure S2), the slight difference in skewness between WT and *xxt1 xxt2* protoplasts may depend on the decrease in mean intensity. These results suggest that the network structure of the primary cell wall shows no significant difference between WT and *xxt1 xxt2* mutant protoplasts, although a decrease in the synthesis of β-glucan fibers was observed in *xxt1 xxt2* protoplasts.

### 2.2. Evidence of Exogenous Xyloglucan Incorporation into the Cell Wall Network during Regeneration from xxt1 xxt2 Protoplasts

**Figure 2:**
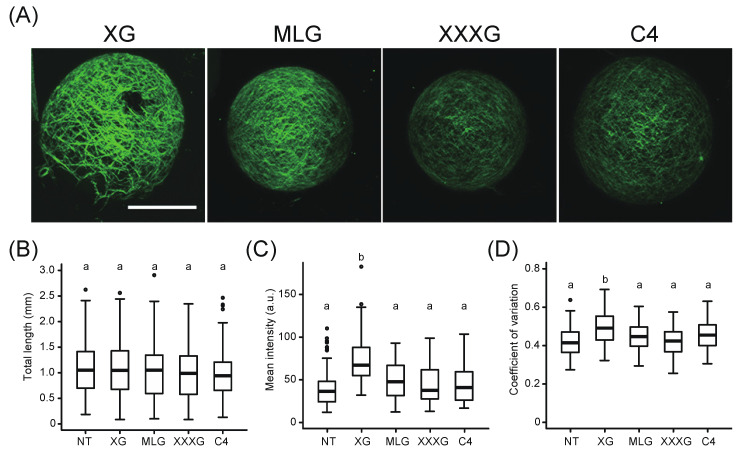
Effect of exogenous application of various polysaccharides or oligosaccharides on cell wall regeneration from *xxt1 xxt2* protoplasts. (**A**) Representative images of Calcofluor-stained fiber regenerated from *xxt1 xxt2* protoplasts incubated in the regeneration medium containing various polysaccharides or oligosaccharides for 24 h. Scale bar = 20 μm. (**B**–**D**) Total length (**B**), mean intensity (**C**), and CV of fluorescence intensity distribution (**D**) of Calcofluor-stained network on the surface of *xxt1 xxt2* protoplasts. NT, no treatment; XG, xyloglucan; MLG, mixed-linkage glucan; XXXG, xyloglucan oligosaccharide; C4, cellotetraose. Center lines of box-plot show the medians, boxes indicate interquartile range (IQR), whiskers indicate 1.5 IQR, and outliers are shown by dots. Significance was determined by Tukey–Kramer test. Values with “a” and “b” are statistically different at *p* < 0.01. *n* = 108 (NT), 112(XG), 109(MLG), 87 (XXXG), and 77 (C4).

To investigate the effects of xyloglucan on cell wall formation, we evaluated the total length, mean intensity, and CV of Calcofluor-stained fibers in the cell wall network structure of *xxt1 xxt2* protoplasts regenerated in the presence or absence of exogenous xyloglucan for 24 h. The addition of xyloglucan resulted in a thick and clear network structure (Figure 2A) and in an increase in the mean intensity and CV values (Figure 2C,D), although no difference was observed in the total length of the network between NT and XG treatment (Figure 2B). We also used another polysaccharide (MLG) and two oligosaccharides (XXXG and C4) as controls because MLG is major hemicellulose of Poales, XXXG is an oligosaccharide obtained by cutting tamarind xyloglucan into tetrasaccharides, and C4 is cellotetraose without a xylose side chain. however, the addition of a polysaccharide or other oligosaccharides did not affect the total length, mean intensity, and CV of intensity distribution (Figure 2). These results suggest that exogenous xyloglucan application altered the fluorescence properties of the Calcofluor-stained cellulose network regenerated from *xxt1 xxt2* protoplasts.

**Figure 3:**
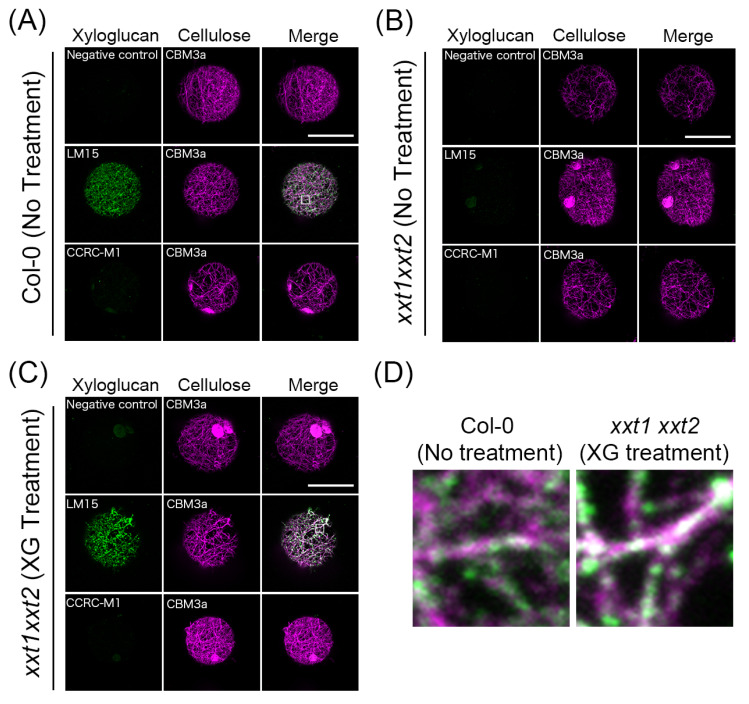
Immunofluorescent labeling of xyloglucan and crystalline cellulose on the surface of Col-0 and *xxt1 xxt2* mutant protoplasts. (**A**–**C**) Immunocytochemistry of protoplasts incubated in the regeneration medium lacking xyloglucan (**A**,**B**) or containing xyloglucan (**C**) for 24 h. Crystalline cellulose was stained with CBM3a, and xyloglucan was stained with LM15 or CCRC-M1. Distilled water was added instead of LM15 or CCRC-M1 as the negative control. (**D**) Magnified images of the white squares in (**A**) (left) and (**C**) (right). These images show the overlap between LM15 and CBM3a signals. Scale bar = 20 μm.

To investigate whether exogenous xyloglucan was incorporated into the regenerated cell wall, we performed immunocytochemistry analysis using xyloglucan- and cellulose fibril-specific monoclonal antibodies: the LM15 antibody, which recognizes XXXG subunits; CCRC-M1 antibody, which recognizes fucosylated side chains of xyloglucans; and CBM3a, which recognizes crystalline cellulose or cellulose fibrils. When WT protoplasts were incubated in the regeneration medium for 24 h, dot-like LM15 signals were observed on the cell surface, and these signals overlapped with cellulose fibrils recognized by CBM3a (Figure 3A,D). On the other hand, little or no signals of CCRC-M1 were detected, indicating that most xyloglucans were not fucosylated in the cell wall regenerated from WT protoplasts. When *xxt1 xxt2* protoplasts were incubated in the regeneration medium for 24 h, CBM3a signals were detected, but neither LM15 signals nor CCRC-M1 signals were detected (Figure 3B). However, dot-like LM15 signals overlapping CBM3a signals were observed in *xxt1 xxt2* protoplasts incubated for 24 h in the regeneration medium containing xyloglucan (Figure 3C,D). This result shows that exogenous xyloglucan was incorporated into the cell wall network regenerated from *xxt1 xxt2* protoplasts.

### 2.3. Effect of Exogenous Xyloglucan on the State of Cellulose Microfibrils during Regeneration of xxt1 xxt2 Protoplasts

**Figure 4:**
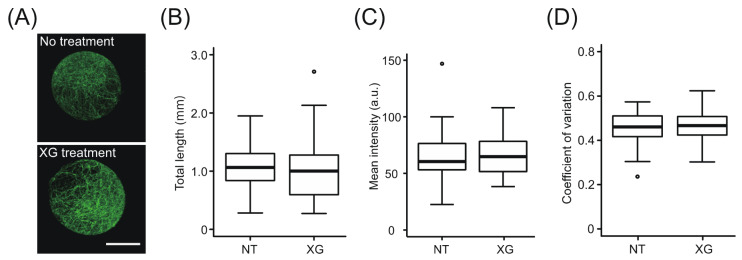
Immunofluorescent labeling of crystalline cellulose on the surface of *xxt1 xxt2* protoplasts. (**A**) Representative images of crystalline cellulose network regenerated from *xxt1 xxt2* protoplasts incubated in the regeneration medium lacking xyloglucan (NT) or containing xyloglucan (XG) for 24 h. Protoplasts were examined by immunocytochemistry using CBM3a antibody, which specifically recognizes crystalline cellulose. (**B**–**D**) Total length (**B**), mean intensity (**C**), and CV of fluorescence intensity distribution (**D**) of CBM3a-labeled network on protoplast surface. Center lines of box-plot show the medians, boxes indicate interquartile range (IQR), whiskers indicate 1.5 IQR, and outliers are shown by dots. Significance (vs. NT) was determined by Mann–Whitney test (*p* < 0.05; *n* = 49 (NT) and 50 (XG)). Scale bar = 20 μm.

To determine whether the change in the Calcofluor-stained network in the presence of xyloglucan was caused by a state change of cellulose microfibrils or by the deposition of xyloglucan, we characterized the fluorescent signals of the network using immunocytochemistry with CBM3a, which specifically recognizes cellulose fibrils (Figure 4A). Image analysis revealed no differences in the values of total length, mean intensity, and CV of intensity distribution between *xxt1 xxt2* protoplasts incubated in the presence of xyloglucan vs. those incubated in the absence of xyloglucan (Figure 4B–D). Although CBM3a is reported to bind to xyloglucan [35], our image analysis did not detect the difference in mean intensity between with or without xyloglucan. This may be due to the lower specificity of CBM3a to xyloglucan compared to cellulose [35]. This result suggests that the increase in mean intensity and CV of Calcofluor-stained network in the presence of xyloglucan was mainly caused by the deposition of xyloglucan.

**Figure 5:**
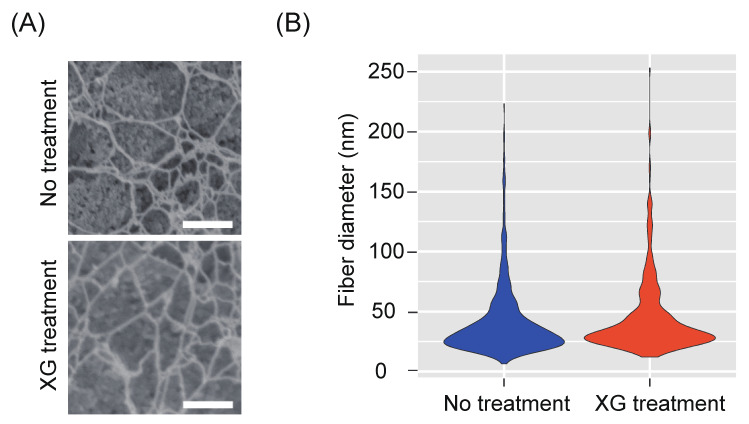
High-resolution imaging analysis of cellulose network, and measurement of fiber diameter in *xxt1 xxt2* protoplasts. (**A**) Field-emission scanning electron microscope (FE-SEM) images of the surface of *xxt1 xxt2* protoplasts incubated in regeneration medium lacking xyloglucan (top) or containing xyloglucan (bottom) for 12 h. (**B**) Violin plot of fiber diameter calculated from the FE-SEM images (*n* = 500 from 50 images). Significance (vs. No treatment) was determined by Student’s *t*-test (*p* < 0.05). Scale bar = 500 nm.

To further investigate the state of cellulose microfibrils regenerated from *xxt1 xxt2* protoplasts, we performed high-resolution imaging analysis of cellulose microfibrils using a field-emission scanning electron microscope (FE-SEM), and measured the cellulose network diameter. The *xxt1 xxt2* double mutant protoplasts were incubated in the regeneration medium for 12 h, and cellulose fibers on the protoplast surface were individually observed. Most of the cellulose microfibrils observed in this network were bundled, and a number of pores were formed by the branching of these bundles (Figure 5A). Fibers with a diameter of 20–30 nm were the most abundant, and the frequency distribution of fiber diameter showed no significant difference between *xxt1 xxt2* protoplasts incubated in the presence of xyloglucan vs. those incubated in the absence of xyloglucan (Figure 5B). This result suggests that xyloglucan does not affect the bundling of cellulose network regenerated from *xxt1 xxt2* protoplasts.

### 2.4. Role of Xyloglucan (XG) in the Osmotic Stability of Protoplasts

**Figure 6:**
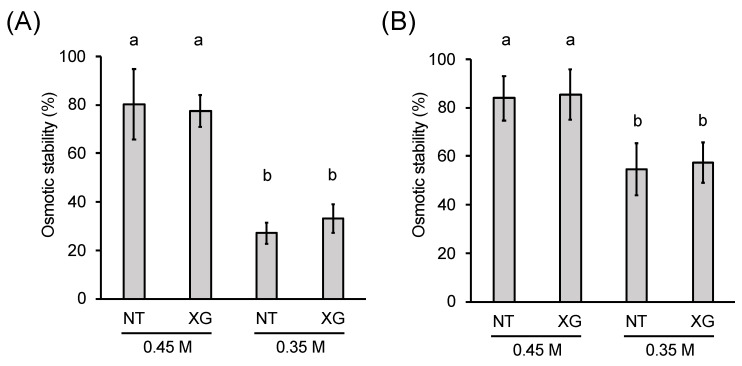
Osmotic stability of *xxt1 xxt2* protoplasts. Osmotic stability of *xxt1 xxt2* protoplasts incubated in the regeneration medium lacking xyloglucan (NT) or containing xyloglucan (XG) for 24 h (**A**) or 48 h (**B**). Protoplasts were incubated in the indicated media for the indicated time points and then transferred to 2-morpholinoethanesulfonic acid (MES) buffer containing either 0.45 M mannitol (same osmotic pressure as the regeneration medium) or 0.35 M mannitol (lower osmotic pressure than the regeneration medium) for 30 min. The ratio of non-ruptured protoplasts after 30 min of incubation was defined as osmotic stability. The experiment was repeated three times using more than 200 cells. Significance was determined by Tukey–Kramer test. Values with “a” and “b” are statistically different at *p* < 0.05.

Xyloglucan may affect the mechanical properties of cell walls without affecting the bundling of cellulose microfibrils. To evaluate the mechanical strength of cell walls regenerated from protoplasts in the presence or absence of xyloglucan, we analyzed the stability of protoplasts against osmotic pressure. The *xxt1 xxt2* protoplasts were incubated in the presence or absence of xyloglucan for 24 h, and then transferred either to 0.45 M mannitol buffer, which has the same osmotic pressure as the regeneration medium, or to 0.35 M mannitol buffer, which exerts lower osmotic pressure than the regeneration medium. After 30 min of incubation, approximately 70% of the protoplasts were ruptured in the 0.35 M mannitol buffer, whereas only 20% of the protoplasts were ruptured in the 0.45 M mannitol buffer (Figure 6A). Importantly, no significant difference was observed in the osmotic stability of *xxt1 xxt2* protoplasts between the two buffers, irrespective of the presence or absence of xyloglucan in the regeneration medium (Figure 6A). Furthermore, pre-incubation of protoplasts in xyloglucan-containing regeneration medium for 48 h did not affect the stability of protoplasts, although the percentage of non-ruptured protoplasts increased to approximately 55%, irrespective of the presence or absence of xyloglucan (Figure 6B). This result clearly indicates that the incorporation of xyloglucan into the cell wall network does not alter the osmotic stability of protoplasts, thus providing direct evidence supporting our finding that the xyloglucan-less cellulose network provides sufficient mechanical strength to the primary cell wall regenerated from protoplasts.

## 3. Discussion

Since the first primary cell wall model proposed by Peter Albersheim and colleagues [5], xyloglucan has been believed to play a pivotal role in the construction and remodeling of the cell wall architecture at least in angiosperms. While multiple lines of evidence indicated that xyloglucan is important for regulating cell wall extensibility during plant growth [6,7,8,9,13,14,23,27], the role of xyloglucan in the formation of the cell wall remains largely unknown. To address this issue, in this study, we employed a new method to objectively evaluate cell wall regeneration from *Arabidopsis* mesophyll protoplasts [32,33]. Using this technique, we acquired image data of cell walls regenerated from WT and *xxt1 xxt2* double mutant protoplasts in the presence or absence of xyloglucan to examine whether or not xyloglucan is required for cell wall construction per se.

Our data showed no remarkable defect in the cellulose network in the absence of xyloglucan. Furthermore, exogenously applied xyloglucan was incorporated into the cell wall network regenerated from *xxt1 xxt2* protoplasts, as visualized by double staining of LM15 and CBM3a, and did not affect the pattern of cellulose network or the diameter of cellulose fibers, as shown by FE-SEM images. These results clearly indicate that the formation of cellulose network structure during the cell wall regeneration process is independent of xyloglucans.

Some studies previously reported that the *xxt1 xxt2* double mutant exhibits disruption of cellulose organization [25,26]. However, this phenotype was not observed in the cell wall regenerated from *xxt1 xxt2* protoplasts in the current study. While we observed nascent cell walls regenerated from protoplasts, in previous reports cell walls in mature tissue such as roots and hypocotyls were studied. Thus, this discrepancy may be a result of differences in the stage of cell wall network formation.

The orientation of cellulose deposition is directly regulated by the orientation of cortical microtubules. In protoplasts, however, cortical microtubules quickly depolymerize within 12 h of incubation after the completion of protoplast preparation [33]. Therefore, it is likely that the initial assembly of cellulose fibrils is not strictly controlled by the orientation of cortical microtubules; instead, cellulose microfibrils might self-assemble via an unknown mechanism during the initial stages of cell wall formation. This consideration is supported by the very recent finding that the trajectory of the cellulose synthase (CESAs) complex is controlled not only by the cortical microtubules but also by the nascent cellulose microfibrils [36]. In particular, under conditions where cortical microtubules do not exist, such as those which occur during cell wall regeneration from protoplasts, this mechanism might function preferentially. Furthermore, another recent finding on the role of CSLDs in synthesizing β-1,4-glucans [37] might imply still unknown mechanism for cellulose deposition independent of a cortical microtubule array in the cell wall regeneration process as well as in the anisotropic cell expansion processes such as tip growth in root hairs and pollen tubes [38,39].

Cell walls from the WT *Arabidopsis* plant have been reported to contain fucosylated xyloglucan [40]. However, our immunocytochemistry with the CCRC-M1 antibody showed that the nascent cell wall regenerated from WT protoplasts contained little or no fucosylated xyloglucan. Since the fucosyl side chains in xyloglucan is presumed to enhance the binding ability to cellulose [41,42], the lack of fucocylation in the xyloglucan in nascent cell wall might imply its less important roles in terms of mechanical integrity of the cellulose network during early stage of cell wall regeneration.

The osmotic stability test showed that the cellulose network in the cell wall regenerated from *xxt1 xxt2* protoplasts can maintain protoplast stability in the absence of xyloglucans, indicating that xyloglucan does not contribute directly to the mechanical strength of the nascent cell wall regenerated from protoplasts. Recently, an atomic force microscopic analysis of the mechanical properties of cell walls in the shoot apical meristem of WT and *xxt1 xxt2* double mutant Arabidopsis plants revealed no difference in Young’s modulus between the two genotypes [43]. This result is consistent with our results of the osmotic stability test. On the other hand, the cell wall of etiolated *xxt1 xxt2* hypocotyls is weaker than that of WT hypocotyls, and *xxt1 xxt2* petioles are more extensible than WT petioles [16,27]. One possible explanation for these contradictory results is that the role of xyloglucan in cell walls of mature petioles and hypocotyls is different from that in the early stage of primary cell wall formation. Clearly, the mechanical environment around nascent cell walls regenerated from protoplasts in suspension culture is quite different from the cell walls within growing tissues of hypocotyls and petioles.

Moreover, the altered mechanical properties of mature tissues of *xxt1 xxt2* mutant plants might reflect a different role of xyloglucan than that evident in *xxt1 xxt2* protoplasts. Xyloglucan has been suggested to be involved in expansin-mediated cell wall expansion [16,27], as well as the XTH-mediated cell-wall remodeling process [13,14,15]. Recent studies showed an important role of xyloglucan in the cell wall loosening which occurs in meristematic tissues of shoot apical meristem [43,44]. Therefore, it is quite likely that roles of xyloglucan during the cell wall regeneration process are distinct from those in the cell wall of dividing and expanding cells.

In conclusion, our results showed that xyloglucan is not essential during the early stages of cellulose network assembly, although it might be required for the maintenance of cell wall mechanics during cell growth. Further research is necessary to understand the effect of xyloglucan on the mechanical properties of the primary cell wall and formation of the cellulose network structure.

## 4. Materials and Methods

### 4.1. Plant Material and Growth Condition

*Arabidopsis thaliana* (L.) Heynh. ecotype Columbia (Col-0) was used as the WT. Seeds of Col-0 and *xxt1 xxt2* double mutant [16] were sown on Rockwool blocks (Grodan, Rock-wool B.V.) moistened with MGRL medium [45]. Seedlings were grown under continuous light conditions (60–70 μmol m^−2^ s^−1^) at 22 °C in a growth chamber.

### 4.2. Protoplast Isolation and Incubation

Mesophyll protoplasts were isolated as described previously [33], with slight modifications. Briefly, fully expanded rosette leaves were detached from 20-day-old *Arabidopsis* plants, sterilized by immersion in 70% ethanol for 30 s, and washed twice with 0.45 M mannitol. Excess buffer was removed using paper towels. Leaves were then cut into strips and immersed in 15 mL of enzyme solution (1% cellulase R-10 and 0.4% macerozyme R-10 [Yakult Pharmaceutical Ind. Co. Tokyo, Japan], 0.45 M mannitol, 20 mM KCl, 10 mM CaCl_2_, and 20 mM MES; pH 5.7) in a Petri dish (9 cm diameter). The immersed specimens were infiltrated under reduced pressure for 5 min, and then incubated at room temperature and atmospheric pressure for 5 h. Protoplasts were isolated from incubated specimens by gentle shaking. An equal volume (15 mL) of W5 solution (154 mM NaCl, 125 mM CaCl_2_, 2 mM KCl, and 2 mM MES; pH 5.7) was added to the protoplast suspension, and the sample was filtered through 100 μm nylon mesh, followed by 50 μm nylon mesh, to remove large tissue debris. The filtered samples were transferred to a 50 mL Falcon tube and centrifuged at 100× *g* for 2 min. The supernatant was removed, and protoplasts were suspended in 10–40 mL of W5 solution. Protoplast density was adjusted to approximately 5 × 10^3^ protoplasts mL^−1^. Then, 5 mL of the suspension was dispensed into a 50 mL Falcon tube and centrifuged at 100× *g* for 2 min. The supernatant was removed as much as possible, and protoplasts were suspended in cell wall regeneration medium (Gamborg’s B-5 basal medium with minimal organics [SIGMA], 0.4 M trehalose dihydrate, 0.05 M glucose, 1 μM 3-naphthalene acetic acid [NAA], and 0.05% MES; pH 5.7) or cell wall regeneration medium containing either 0.05% tamarind xyloglucan (P-XYGLN, Megazyme), barley β-glucan (P-BGBH, Megazyme), xyloglucan heptasaccharide (O-X3G4, Megazyme), or cellotetraose (O-CTE-100MG, Megazyme). The protoplast suspension was incubated for 6, 12, 18, 24 or 48 h under continuous light conditions (60–70 μmol m^−2^ s^−1^) at 22° C in a growth chamber.

### 4.3. Calcofluor Staining and Image Acquisition

Protoplasts were incubated in regeneration medium containing 0.001% Calcofluor (SIGMA) for 5 min and then washed once in regeneration medium. Calcofluor signals were observed under a confocal laser scanning microscope (FV-1000-D, Olympus) using a ×100 oil immersion objective lens (NA = 1.30). Fluorescent images were acquired with a 405 nm laser, a DM405/473/559/635 dichroic mirror and a 430–455 nm emission filter. Serial optical sectional images from the top to the center of the protoplast were acquired at 0.5 μm resolution. Fifty protoplast images were acquired per experiment and were repeated three times. Then, the following image analysis was performed using the image in which the regenerated cell wall was observed.

### 4.4. Image Analysis

To quantitatively analyze the Calcofluor-stained or CBM3a-stained cellulose network, maximum intensity projection images obtained from the serial optical sectional images were skeletonized using the ImageJ plugin LpxLineExtract, which is invoked by the Lpx_Filter2d plugin in the LPixel ImageJ plugins package [46]. Parameters used for line extraction were as follows: giwsIter = 2; mdnmsLen = 3; pickup = above; shaveLen = 5; delLen = 5. The area excluding the region of interest, such as background signals and callose-like dot signals, was masked by manual segmentation [33]. Total length, mean intensity, CV, and skewness were measured from the masked images using the ImageJ plugin LpxLineFeature, which is invoked by the Lpx_Filter2d plugin in the LPixel ImageJ plugins package. Total length estimates the apparent amount of the network on the protoplast surface, and is calculated by converting the number of pixels constituting cellulose network (*N_cellulose_*) to millimeters (mm). Mean intensity ($\bar{i}$) represents the mean fluorescent intensity of Calcofluor or CBM3a signals. The CV is a measure of the dispersion of intensity distribution. When cellulose fibrils are bundled, a region with high fluorescence intensity appears partially, so the intensity distribution is expected to extend to the high-intensity side. This leads to an increase in the dispersion of intensity distribution, which increases CV. The CV is obtained by dividing the standard deviation of fluorescence intensity by the mean value of fluorescence intensity. Skewness (S) is a measure of the asymmetry of intensity distribution. When cellulose fibrils are bundled, a region with high fluorescence intensity appears partially, so the intensity distribution is expected to extend to the high intensity side. This leads to an increase in the asymmetry, which increases S [33,47]. S was calculated using the following equation:

(1) $$S=\frac{1}{N_{cellulose}}\overset{N_{cellulose}}{\underset{i=1}{\sum }}{\left(\frac{i_{n}-\bar{i}}{\sigma }\right)}^{3}$$

(2) $$\sigma =\frac{1}{N_{cellulose}}\overset{N_{cellulose}}{\underset{i=1}{\sum }}{\left(i_{n}-\bar{i}\right)}^{2}$$

*N_cellulose_*, $\bar{i}$, *CV*, and *S* were measured as i_nPix, i_mean, i_stddevPerMean, and i_skewness, respectively, using the LpxLineFeature ImageJ plugin.

### 4.5. Immunocytochemistry

To stain cellulose using the CBM3a antibody, incubated protoplasts were transferred to the blocking solution (0.1% MES, 0.45 M trehalose, and 1% TSA blocking reagent [Molecular Probes]; pH 5.7) and incubated for 30 min in darkness. The blocking solution was removed, and protoplasts were incubated with CBM3a primary antibody (1:100 dilution [*v*/*v*]) in blocking solution for 2 h in darkness. Protoplasts were then washed three times with wash buffer (0.45 M trehalose and 0.1% MES; pH 5.7), and incubated with rabbit anti-His-tag polyclonal antibody (MBL, Nagoya, Japan) (1:100 dilution [*v*/*v*]) in blocking solution for 1 h in darkness. Protoplasts were then washed three times with wash medium and incubated with anti-rabbit IgG Alexa Flour 488 secondary antibody (1:50 dilution [*v*/*v*]) in blocking solution for 2 h in darkness. Protoplasts were then washed three times with wash medium, and images were acquired under a confocal laser scanning microscope (FV-1000-D, Olympus) using a 473 nm laser.

Double staining of xyloglucan and cellulose was performed according to the same procedure as described above, except a mixture of CBM3a (1:100 dilution [*v*/*v*]) with either LM15 or CCRC-M1 (1:10 dilution [*v*/*v*]) was used as the primary antibody, and a mixture of anti-rat IgG Alexa Flour 488 and anti-rabbit IgG Alexa Fluor 594 (1:50 dilution [*v*/*v*]) was used as the secondary antibody. Images were acquired under a confocal laser scanning microscope (FV-1000-D, Olympus) using a 473 nm laser for LM15 or CCRC-M1, and 559 nm laser for CBM3a. In this study, LM15 and CBM3a were supplied by [48], and CCRC-M1 was supplied by [49].

### 4.6. Field-Emission Scanning Electron Microscopy (FE-SEM)

The incubated protoplasts were washed three times with wash buffer (0.05% MES and 0.45 M mannitol; pH 5.7) and fixed in 2.5% glutaraldehyde in wash buffer at 4° C for 30 min. After washing three times with wash buffer, post-fixation was carried out with 1% osmium tetroxide in wash buffer at 4° C for 30 min. To monitor pectin degradation, the fixed protoplast specimens were washed three times with distilled water and incubated in pectate lyase buffer (10 μg/mL pectate lyase [E-PLYCJ, Megazyme], 50 mM CAPS (3-(cyclohexylamino)-1-propanesulfonic acid) buffer, 2 mM CaCl_2_, 2 mM NaN_3_; pH 10) at room temperature for 40 h. Dehydration was carried out using a graded ethanol series (50%, 70%, 80%, 90%, and 100% three times) for 10 min each. The specimens were transferred to t-butyl alcohol three times for 5 min each and freeze-dried using a freeze dryer (ES-2030, Hitachi, Tokyo). The freeze-dried samples were sputter coated twice with platinum palladium using a Magnetron sputter (MSP-1S, VACUUM DEVICE, Ibaraki) and then secondary electron images were acquired using a field-emission scanning electron microscope (FE-SEM; SU8000, Hitachi, Tokyo) (working distance = ~13 mm, accelerating voltage = 30 kV).

### 4.7. Osmotic Stability Test

Protoplasts incubated in regeneration medium supplemented with or without xyloglucan were transferred to 0.05% MES buffer containing 0.45 M mannitol (pH 5.7) three times at 5 min intervals. Before changing the osmotic pressure, the number [N_0_] of non-ruptured protoplasts was measured using a portion of the cells. Subsequently, the remaining protoplasts were transferred to 0.05% MES buffer containing 0.45 M or 0.35 M mannitol (pH 5.7) and incubated for 30 min, and then the number [N_30_] of non-ruptured protoplasts was measured. The ratio of non-ruptured protoplasts after 30 min of incubation was calculated by dividing [N_30_] by [N_0_], and was designated as osmotic stability.

## Supplementary Materials

The following are available online at https://www.mdpi.com/2223-7747/9/5/629/s1: Figure S1: Changes in coefficient of variation (CV) of fluorescence intensity distribution of Calcofluor-stained network in the Taxol treatment during cell wall regeneration, Figure S2: Correlation of mean intensity with skewness and CV of the regenerated cellulose network in protoplasts incubated in the regeneration medium for 24 h.
