# Supplementary material for: Xyloglucan Is Not Essential for the Formation and Integrity of the Cellulose Network in the Primary Cell Wall Regenerated from Arabidopsis Protoplasts
Source: Plants (Basel). 2020 May 14;9(5):629. doi: 10.3390/plants9050629 (PMC7285283; doi:10.3390/plants9050629)
Supplement: Supplementary file 1 [file plants-09-00629-s001.pdf]

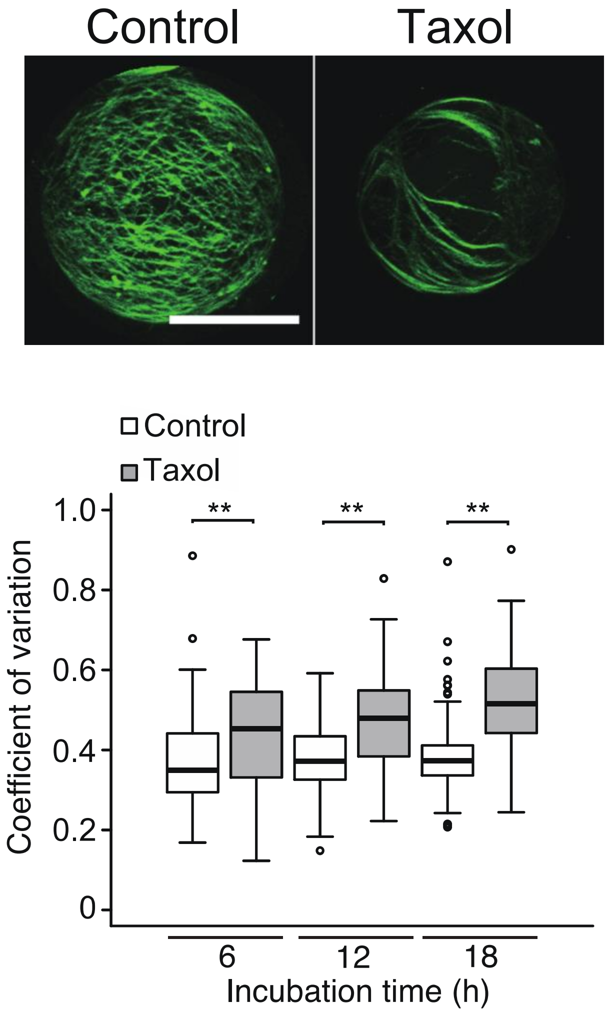

Figure S1. Changes in coefficient of variation (CV) of fluorescence intensity distribution of Calcofluor-stained network in the Taxol treatment during cell wall regeneration. The wild-type protoplasts were incubated for 6, 12, or 18 h in the presence (Taxol) or absence (Control) of 10 μM Taxol, and cell wall network was stained with calcofluor. Representative images of Calcofluor-stained network structure at 18 h of incubation were shown (top), and CV of fluorescence intensity distribution was measured (bottom). This figure was created based on the data acquired in [33]. Center lines of box-plot show the medians, boxes indicate interquartile range (IQR), whiskers indicate 1.5 IQR, and outliers are shown by dots. Significance was determined by Mann-Whitney test. **; $p < 0.01$. n = 123 (6 h), 111 (12 h), 125 (18 h) in Control condition, and 112 (6 h), 104 (12 h), 132 (18 h) in Taxol condition. Scale bar = 20 μm.

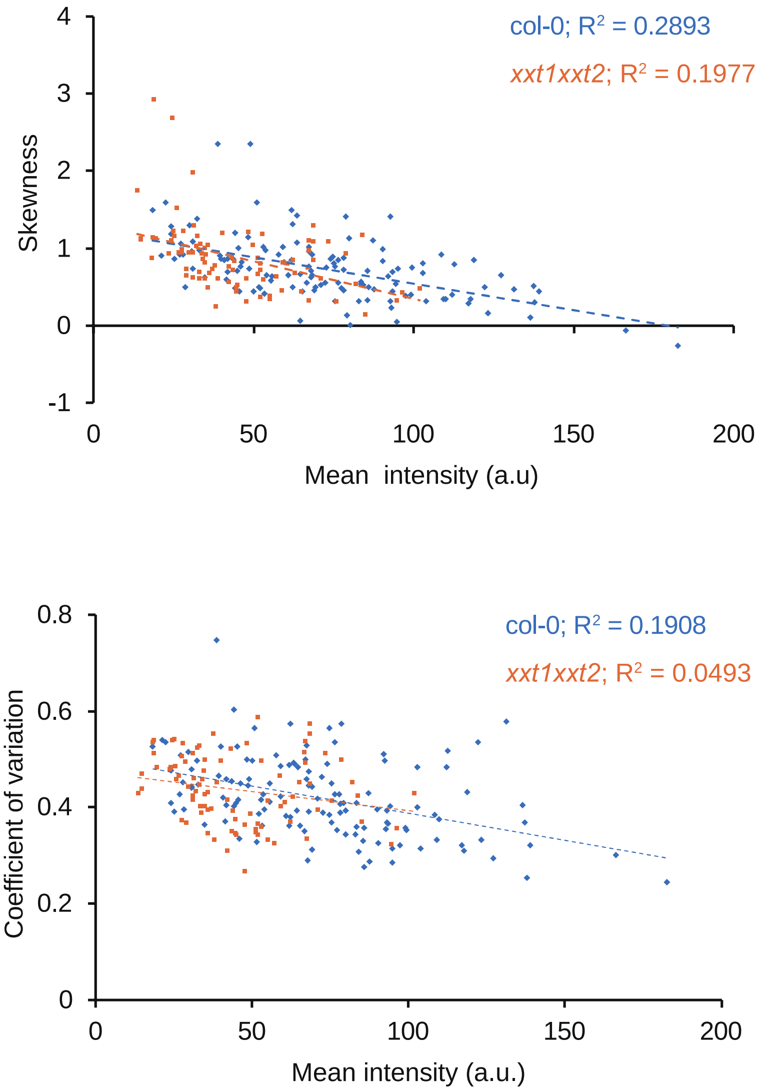

Figure S2. Correlation of mean intensity with skewness and CV of the regenerated cellulose network in protoplasts incubated in the regeneration medium for 24 h. Protoplasts were incubated for 24 h, and scatter plot of mean intensity versus skewness (**A**) or CV (**B**) of Calcofluor-stained network were drawn. n = 130 (Col-0) and 88 (*xxt1 xxt2*).
